# Sumoylation regulates the stability and nuclease activity of *Saccharomyces cerevisiae* Dna2

**DOI:** 10.1038/s42003-019-0428-0

**Published:** 2019-05-08

**Authors:** Lepakshi Ranjha, Maryna Levikova, Veronika Altmannova, Lumir Krejci, Petr Cejka

**Affiliations:** 1 Institute for Research in Biomedicine, Università della Svizzera italiana (USI), Faculty of Biomedical Sciences, Via Vincenzo Vela 6, 6500 Bellinzona, Switzerland; 2 Institute of Molecular Cancer Research, University of Zürich, Winterthurerstrasse 190, 8057 Zürich, Switzerland; 3 Department of Biology, Masaryk University, Kamenice 5, 625 00 Brno, Czech Republic; 4 International Clinical Research Center, St. Anne’s University Hospital, 656 91 Brno, Czech Republic; 5 National Center for Biomolecular Research, Masaryk University, Kamenice 5, 625 00 Brno, Czech Republic; 6 Department of Biology, Institute of Biochemistry, Eidgenössische Technische Hochschule (ETH), 8093 Zürich, Switzerland

**Keywords:** Genomic instability, DNA

## Abstract

Dna2 is an essential nuclease-helicase that acts in several distinct DNA metabolic pathways including DNA replication and recombination. To balance these functions and prevent unscheduled DNA degradation, Dna2 activities must be regulated. Here we show that *Saccharomyces cerevisiae* Dna2 function is controlled by sumoylation. We map the sumoylation sites to the N-terminal regulatory domain of Dna2 and show that in vitro sumoylation of recombinant Dna2 impairs its nuclease but not helicase activity. In cells, the total levels of the non-sumoylatable Dna2 variant are elevated. However, non-sumoylatable Dna2 shows impaired nuclear localization and reduced recruitment to foci upon DNA damage. Non-sumoylatable Dna2 reduces the rate of DNA end resection, as well as impedes cell growth and cell cycle progression through S phase. Taken together, these findings show that in addition to Dna2 phosphorylation described previously, Dna2 sumoylation is required for the homeostasis of the Dna2 protein function to promote genome stability.

## Abstract (web summary)

Ranhja et al. find that budding yeast Dna2 is modified by sumoylation, and that sumoylation reduces the nuclease, but not helicase activity of Dna2 in vitro. In cells, expression of a Dna2 version that cannot be sumoylated leads to impaired DNA end resection and cell division.

## Introduction

Post-translational modifications (PTMs) regulate protein functions through multiple mechanisms. The covalent attachment of a small ubiquitin-like modifier (SUMO) to target substrates is a common modification with a widespread role in DNA damage response^1–4^. SUMO is a small, 76 amino acids long peptide, which is attached to a lysine residue of the target substrate via its C-terminal glycine. These enzymatic reactions are catalyzed by E1, E2, and E3 ligases, similarly to the ubiquitination system. While vertebrate cells possess three SUMO proteins (SUMO1 and very similar SUMO2 and SUMO3), *Saccharomyces cerevisiae* contain only a single SUMO protein named Smt3^1,5^.

Sumoylation may compete with other PTMs, such as ubiquitination, acetylation, methylation, hydroxylation, or poly ADP-ribosylation. It may thus affect protein function indirectly by interfering with other regulatory pathways, or have a more direct effect. Sumoylation can positively or negatively regulate protein stability and proteasomal degradation^6^. Additionally, sumoylation can affect protein–protein interactions, binding of proteins to nucleic acids, recruitment and subcellular localization or directly regulate enzymatic properties^1–4,7^. A number of proteins can specifically interact with sumoylated proteins via their SUMO-interacting motifs (SIMs)^8^. Notably, the SUMO–SIM interaction can be intermolecular but also intramolecular, as most proteins targeted by sumoylation also bear SIMs^2^. Many proteins acting in DNA metabolism are regulated by sumoylation, although typically only a minor fraction of the protein pool is modified^7^. Proteins involved in the homologous recombination (HR) pathway and DNA damage checkpoint were shown to be subjects of a sumoylation wave following DNA end resection upon DNA damage, but the effects on the activities of the individual proteins remain largely uncharacterized^9^.

Dna2 is an essential nuclease-helicase involved in several key processes of DNA metabolism, including DNA replication, HR and checkpoint activation^10–15^. All Dna2 functions, except for its role in checkpoint activation, absolutely require its nuclease activity. Specifically, the Dna2 nuclease is essential for the processing of long flaps that arise during lagging strand synthesis in DNA replication, while short flaps are mostly processed by flap endonuclease 1 (FEN1/Rad27)^12^. Dna2 also functions in a poorly defined pathway upon replication stress, and may be involved in the degradation of reversed replication forks^16–18^. In addition to DNA replication, Dna2 nuclease functions in conjunction with a cognate RecQ family helicase (Sgs1 in yeast) to resect 5′-terminated DNA end near DNA double-strand breaks (DSBs), producing 3′-terminal ssDNA overhangs at break ends^19^. The helicase activity of Dna2 has a supporting function in this process to degrade ssDNA, while unwinding of dsDNA ahead of Dna2 is catalyzed by Sgs1^14,20–22^. DNA end resection initiates and commits DSB repair to HR. Dna2 is however not the only nuclease that functions in DNA end resection. The Mre11 nuclease within the Mre11-Rad50-Xrs2 complex functions upstream of Dna2 in conjunction with Sae2 and also has a structural role to promote the Sgs1-Dna2 pathway^14,19,21^. The Exo1 nuclease instead functions in parallel with Dna2^19,23,24^. Although most reports suggest that Dna2 and Exo1 represent separate and sometimes redundant pathways, there is evidence that both branches can cooperate in some cases^19,24–26^.

The recruitment of Dna2 to DSBs is stimulated by CDK-dependent phosphorylation, which also promotes DNA end resection^25^. However, the underlying mechanisms that regulate Dna2 levels and activity remain undefined. To date, it has been reported that sumoylation of Sae2 and the Mre11-Rad50-Xrs2 complex promotes DNA resection by limiting inhibitory aggregation^27^. Yeast Mre11 has the capacity to bind sumoylated proteins, which likely facilitates resection complex assembly^28,29^. In contrast, sumoylation of human EXO1 was shown to facilitate its degradation by promoting ubiquitination^30^. Interestingly, ssDNA generated in course of DNA end resection is required for the induction of sumoylation upon DNA damage, showing that DNA end resection proteins are both triggers and targets of sumoylation^9^.

Here, we report that *S. cerevisiae* Dna2 is sumoylated. Sumoylation specifically attenuates the nuclease activity of recombinant Dna2, while the helicase activity is not affected. In cells, sumoylation leads to reduced levels of the total Dna2 protein, indicating that it facilitates Dna2 degradation. However, the non-sumoylatable Dna2 variant shows impaired nuclear localization and reduced recruitment to DNA damage foci. Our results demonstrate the necessity for precise regulation of Dna2 functions to maintain genome stability.

## Results

### *Saccharomyces cerevisiae* Dna2 is sumoylated in vivo by Siz2

**Fig. 1:**
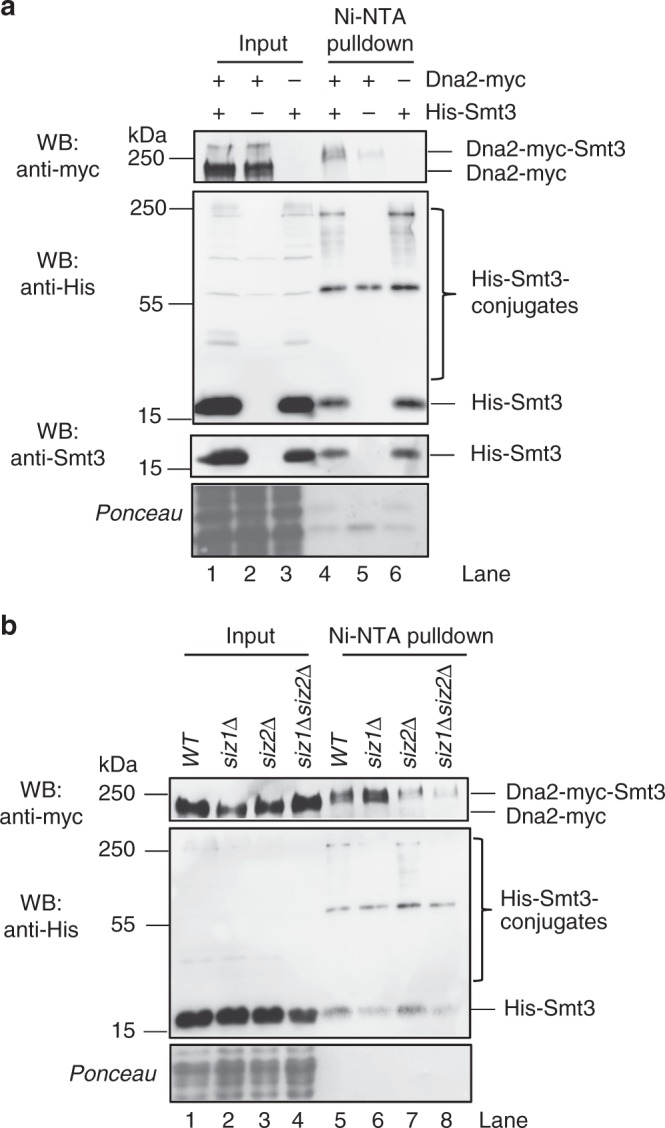
*S. cerevisiae* Dna2 is sumoylated in vivo by the Siz2 SUMO ligase. **a** Ni-NTA pulldown of His-Smt3 (SUMO) protein conjugates from *S. cerevisiae* (FF18733 background) cells expressing myc-tagged Dna2. Cells were transformed with vector coding for His-Smt3 or an empty vector as a negative control. Input and pulldown samples were analyzed by western blotting (WB) using antibodies specific for myc tag (detecting Dna2-myc), Smt3 (detecting SUMO/Smt3) and His tag (detecting all His-tagged Smt3 conjugates). Ponceau-stained membrane sections shown serve as a control for equal loading. A representative western blot is shown. **b** A representative experiment as in **a**, but using wild type, *siz1*Δ, *siz2*Δ and *siz1*Δ *siz2*Δ strains

Multiple factors involved in DNA metabolism are sumoylated, which may regulate their recruitment, stability or biochemical function^4,7^. To test whether *S. cerevisiae* Dna2 is sumoylated, we overexpressed His-tagged Smt3, coding for the yeast SUMO protein. We note that the amount of His-tagged Smt3 is much higher than that of endogenous Smt3, which improves the detection limit^31^. Next, we performed Ni-NTA affinity pulldowns of His-Smt3-protein conjugates under denaturing conditions (Fig. 1), as used previously for other proteins^31,32^. To detect Dna2 in the Ni-NTA eluates, we constructed a *S. cerevisiae* strain expressing Myc-tagged Dna2 from its native chromosomal locus. We were able to identify a slower-migrating Dna2-myc species in the Smt3 pulldown sample (Fig. 1a, lane 4). In contrast, this band was reduced when the Ni-NTA affinity purification was performed with cells transformed with the empty vector that did not express His-Smt3 (Fig. 1a, compare lanes 4 and 5), or when Dna2 was not Myc-tagged (Fig. 1a, compare lanes 4 and 6). This suggested that the slower-migrating Dna2 variant may represent a sumoylated form of Dna2. Since Siz1 and Siz2 are the main SUMO E3 ligases in *S. cerevisiae*^2^, we wished to determine their role in Dna2 sumoylation. We deleted them individually or in combination and repeated the pulldown of the Smt3-protein conjugates (Fig. 1b). Only the deletion of *SIZ2* or both *SIZ1* and *SIZ2*, but not *SIZ1* alone, led to the decrease of the slower-migrating Dna2 signal (Fig. 1b, lanes 5–8). This provided further support that the observed band indeed corresponds to sumoylated Dna2, and identified Siz2 as the main SUMO ligase responsible for this modification.

Next, we set out to determine whether Dna2 sumoylation levels vary throughout the cell cycle. To this point, we synchronized *S. cerevisiae DNA2*-myc cells in the G1 phase by α-factor and monitored Dna2 protein, as well as its sumoylation levels at various time points after release (Supplementary Fig. 1a, b). Surprisingly, the total amounts of Dna2 were low in G1 and early S phase, while they substantially increased in late S and G2/M phases of the cell cycle (Supplementary Fig. 1a, b, see also below). Interestingly, proteasomal degradation of Dna2 upon genotoxic treatments was reported in yeast cells treated with caffeine, independently of its checkpoint inhibition effect^33^. The Dna2 levels throughout the cell cycle differed from those of Rad27, which were highest in early S-phase (Supplementary Fig. 1c). Sumoylation of Dna2 was apparent at the cell cycle phases when Dna2 was abundant (Supplementary Fig. 1a, b). The fluctuation of Dna2 levels throughout the cell cycle might be at least in part due to protein degradation by proteasome. Therefore, we treated asynchronous cells with the proteasomal inhibitor MG132 (or dimethyl sulfoxide, DMSO,  as a control), and added α-factor to synchronize the cells in G1. Dna2 protein levels were then followed by western blotting (Supplementary Fig. 1d–f). The Dna2 protein levels in cells treated with DMSO, relative to α-tubulin, decreased in course of the synchronization as cells were arresting in G1 (Supplementary Fig. 1d–f). In contrast, Dna2 levels were modestly stabilized in cells treated with MG132 (Supplementary Fig. 1d–f) showing that proteasomal degradation may be at least in part responsible for the fluctuation of the Dna2 levels throughout the cell cycle. Finally, we observed that the apparent Dna2 sumoylation level was reduced upon treatment of cells with various DNA-damaging drugs (Supplementary Fig. 1g). However, these drugs are also expected to arrest a proportion of the cells in early S-phase due to problems with DNA replication, as shown for MMS (Supplementary Fig. 1h, i). Therefore, the reduced sumoylation of Dna2 upon DNA damage may simply reflect the reduced overall pool of Dna2.

We note that under all tested conditions, the proportion of sumoylated Dna2 is very low. We failed to detect sumoylated Dna2 in cells that were not transformed with His-tagged Smt3, or without performing the Smt3 pulldown first, as observed with a variety of other proteins^34–36^. In summary, we show here that *S. cerevisiae* Dna2 is likely sumoylated in a reaction dependent on the Siz2 SUMO ligase. Additionally, we found that the Dna2 protein levels vary throughout the cell cycle, with the highest amounts detected at a late S-G2 phase.

### Sumoylation attenuates the nuclease activity of Dna2

**Fig. 2:**
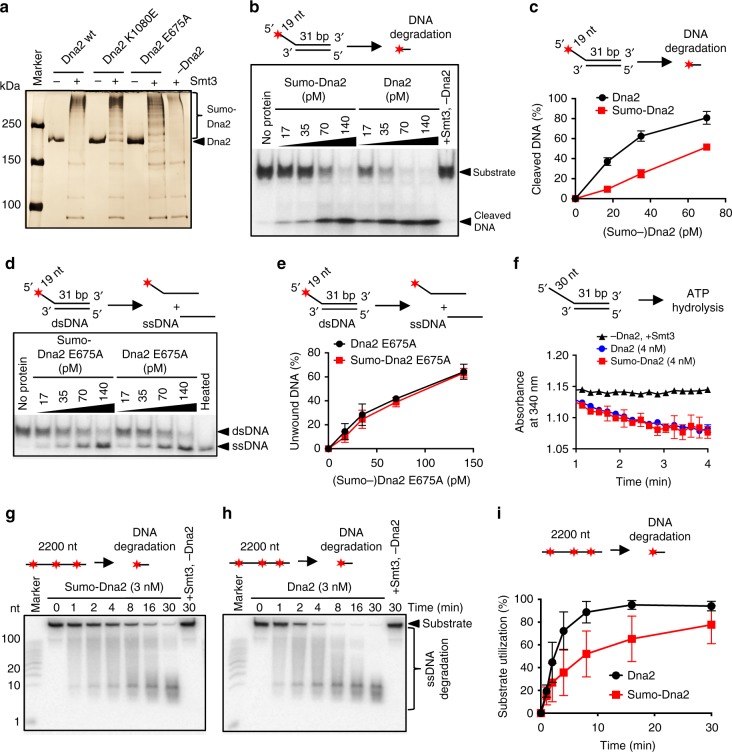
Sumoylation of Dna2 attenuates its nuclease but not its helicase activity in vitro. **a** Silver-stained polyacrylamide gel showing in vitro sumoylated (+) and mock-treated (−) *S. cerevisiae* recombinant Dna2 proteins. Sumoylated Dna2 migrates higher in the gel and is indicated. Dna2 K1080E and Dna2 E675A represent helicase and nuclease-dead variants, respectively. **b** Representative native polyacrylamide gel showing the degradation of 5′-tailed substrate by sumoylated (Sumo-Dna2) or mock-treated Dna2. The top oligonucleotide was ^32^P-labeled at the 5′-end. The reactions contained replication protein A (RPA) and various Dna2 variant concentrations, as indicated. Filled star indicates position of ^32^P label. **c** Quantitation of experiments such as in **b**. Average shown, *n* = 2; whiskers, range. **d** Representative native polyacrylamide gel showing the unwinding of 5′-tailed substrate by sumoylated nuclease-dead Dna2 (Sumo-Dna2 E675A) and mock-treated Dna2 E675A. **e** Quantitation of experiments such as in **d**. Averages shown, *n* = 2; whiskers, range. **f** Quantitation of kinetic experiments showing the absorbance at 340 nm, which corresponds to ATP hydrolysis rate by wild-type sumoylated (Sumo-Dna2) or mock-treated Dna2 (Dna2). Averages shown, *n* = 2; whiskers, range. **g** Representative denaturing polyacrylamide gel showing nucleolytic degradation of randomly labeled 2200 nt-long ssDNA by wild-type sumoylated Dna2 (Sumo-Dna2) in the presence of RPA. **h** Experiment as in **g**, but with non-sumoylated Dna2. **i** Quantitation of experiments such as in **g** and **h**. Shown is the total substrate utilization. Averages shown, *n* = 2; whiskers, range

We next set out to determine the impact of sumoylation on the nuclease and helicase activities of recombinant Dna2. To this point, we first optimized an in vitro sumoylation assay with purified Dna2 and the recombinant sumoylation machinery from *S. cerevisiae* (Fig. 2a). When Smt3 was included in the sumoylation reaction, wild-type Dna2, the nuclease-deficient Dna2 E675A and helicase-deficient Dna2 K1080E polypeptides migrated higher on a polyacrylamide gel compared to the mock-treated (without Smt3) Dna2 variants (Fig. 2a), showing that Dna2 can be efficiently sumoylated in vitro. We next tested the effect of Dna2 sumoylation on its nuclease and helicase activities, using a 5′-tailed oligonucleotide-based DNA substrate (Fig. 2b–e, and Supplementary Fig. 2a, b). Sumoylated wild-type Dna2 was ~3-fold less efficient in substrate cleavage than its non-sumoylated variant from mock reactions lacking Smt3 (Fig. 2b, c). This was similar to helicase-dead Dna2 K1080E (Supplementary Fig. 2a, b), indicating that sumoylation inhibits the nuclease activity of Dna2. The effect was specific to Dna2 sumoylation, as recombinant Smt3 alone had no inhibitory effect on Dna2 nuclease activity when added to the nuclease reactions (Supplementary Fig. 2c).

In order to test the effect of sumoylation on the helicase activity, we utilized the nuclease-deficient Dna2 E675A, as it is established that the DNA unwinding activity of Dna2 can only be observed upon inactivation of the nuclease^37–39^. Using Dna2 E675A, we observed that the unwinding capacity was unaffected by sumoylation (Fig. 2d, e), despite the levels of sumoylation of all Dna2 variants were comparable (Fig. 2a). This suggested that sumoylation specifically inhibits the nuclease, but not the helicase capacity of Dna2. To further confirm this observation, we also analyzed the Dna2 ATPase activity, which is detectable in both wild-type and nuclease-deficient variants. Importantly, sumoylation did not affect the rate of ATP hydrolysis by Dna2 (Fig. 2f), confirming the selective inhibition of the Dna2 nuclease by sumoylation. Previously, we and others proposed that the motor activity of Dna2 may not function as a DNA helicase to unwind dsDNA, but rather as a ssDNA translocase to facilitate degradation of ssDNA produced by Sgs1 during DNA end resection^20,22^. The interplay of the helicase and nuclease activities determines the length of DNA fragments resulting from ssDNA processing: helicase of Dna2 extended the average length of the DNA products^20,22^. To this point, we observed that sumoylation moderately decreased the overall rate of ssDNA degradation by Dna2, in agreement with its inhibitory effect on the Dna2 nuclease (Fig. 2g–i). We also observed that sumoylation of Dna2 led to the generation of longer fragments during the degradation of ssDNA (Supplementary Fig. 2d). The sumoylation of Dna2 instead did not notably affect its DNA-binding activity (Supplementary Fig. 2e–g). In summary, our results demonstrate that sumoylation selectively inhibits the nuclease, but not ATPase, DNA helicase or DNA-binding activities of Dna2 in vitro.

### Dna2 is sumoylated at its N-terminus

**Fig. 3:**
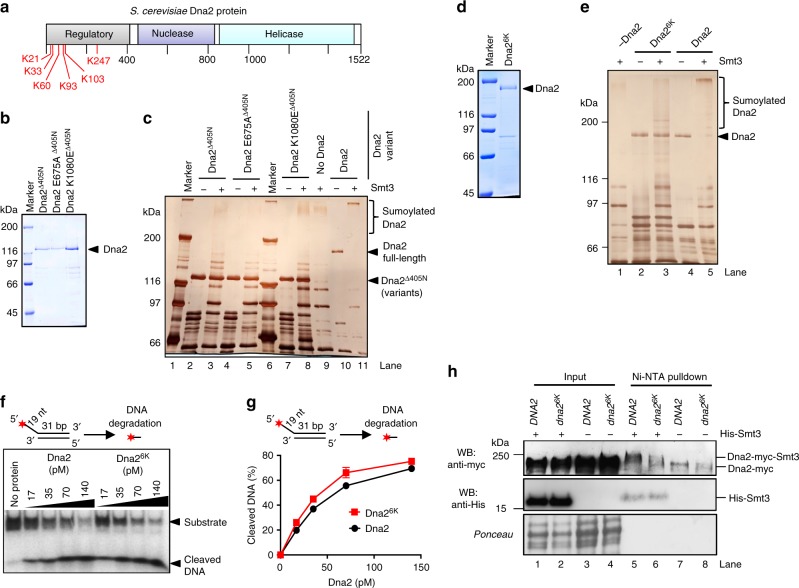
Sumoylation targets the N-terminal regulatory domain of Dna2. **a** A schematic representation of Dna2 domain structure. Sumoylated lysine (K) residues, as determined by mass spectrometry analysis of recombinant Dna2, are depicted in red. **b** Coomassie stained polyacrylamide gel showing purified wt, E675A and K1080E Dna2 variants lacking 405 N-terminal amino acids (Δ405N). **c** Silver-stained polyacrylamide gel showing in vitro sumoylated (+) and mock-treated (−) full-length and Δ405N Dna2 variants. **d** Coomassie stained gel showing recombinant non-sumoylatable Dna2^6K^ (K21A, K33A, K60A, K93A, K103A, K247A). **e** Silver-stained polyacrylamide gel showing in vitro sumoylated (+) and mock-treated (−) wt Dna2 and Dna2^6K^ polypeptides. **f** Representative native polyacrylamide gel showing the degradation of 5′-tailed substrate by wt Dna2 or Dna2^6K^ proteins. g Quantitation of experiments such as in **f**. Averages shown, *n* = 2; whiskers, range. **h** Ni-NTA pulldown of His-Smt3 protein conjugates from wild-type Dna2 and Dna2^6K^ expressing strains (JKM139 background). Representative western blot (WB) from three independent experiments is shown. The bands in the anti-myc western blot in lanes 7 and 8 likely represent non-sumoylated myc-tagged Dna2 that was nonspecifically bound to the Ni-NTA resin

To identify the lysine residues within Dna2 that are sumoylated, we performed mass spectrometry analysis of the in vitro sumoylated sample, and identified six potential sites of modification (Fig. 3a). All these lysines (K21, K33, K60, K93, K103, and K247) map to the unstructured N-terminal domain of Dna2 that was shown to mediate checkpoint activation upon replication stress, facilitate nuclear localization, promote binding to secondary DNA structures, participate in the interaction with RPA and limit the Dna2 nuclease^15,25,37,40,41^. To verify that sumoylation targets the N-terminal domain of Dna2, we tested wild type, nuclease- and helicase-deficient Dna2 variants lacking the N-terminal 405 amino acids in the in vitro sumoylation assay (Fig. 3b, c). The sumoylation of the Dna2^Δ405N^ variants was much less efficient than that of the full-length protein (Fig. 3c, compare fractions of unmodified Dna2 variants upon adding Smt3). This confirmed the N-terminal domain as a target of sumoylation in Dna2. The truncated Dna2 variants were shown to display elevated levels of nuclease activity compared to the full-length protein, implicating the N-terminal domain as an inhibitor of its nuclease activity^41^. Our results thus suggest that sumoylation of the N-terminal domain may strengthen this inhibitory function.

Next, we substituted all six lysines for non-sumoylatable residues and purified the mutant polypeptide (Dna2^6K^). The sumoylation of this recombinant Dna2 variant was notably reduced in comparison to the wild-type protein (Fig. 3d, e, compare lanes 3 and 5, note fractions of unmodified Dna2 variants), confirming the mass spectrometry analysis. Importantly, the nuclease activity of Dna2^6K^ was very similar to that of the wild-type protein, showing that the lysine substitutions per se did not compromise the function of the N-terminal domain to limit the Dna2 nuclease (Fig. 3f, g). To determine whether the six lysine residues are the sites of sumoylation also in vivo, we constructed a myc-tagged *dna2*^*6K*^ strain by allelic replacement strategy^42^. Using this mutant strain and myc-tagged *DNA2* cells as a control, we observed that sumoylation of the Dna2 variant was greatly reduced upon lysine replacement (Fig. 3h, compare lanes 5 and 6). In summary, we identified six lysine residues within the N-terminal regulatory domain of Dna2 that are sumoylated both in vitro and in vivo. Sumoylation of this N-terminal domain appears to strengthen its capacity to limit the nuclease activity of Dna2.

### Non-sumoylatable Dna2 shows impaired recruitment

**Fig. 4:**
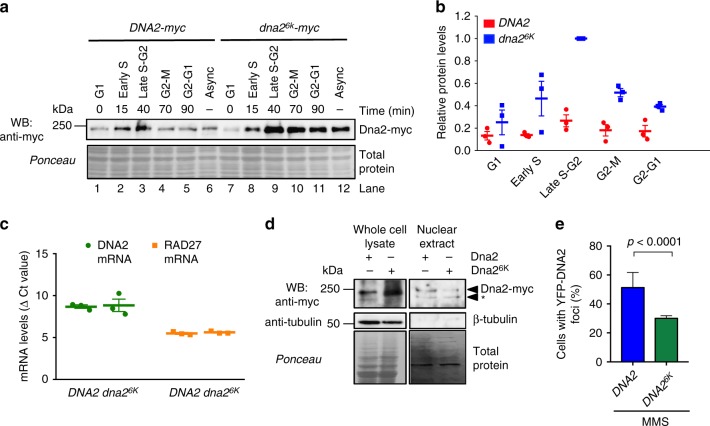
Sumoylation targets Dna2 for degradation. **a** A representative western blot comparing the levels of Dna2 vs. non-sumoylatable Dna2^6K^ proteins. Cells (JKM139 background) were synchronized at G1, and released for indicated times. Ponceau-stained membrane is a control for equal loading. Protein levels from asynchronous cells (Async) are also shown. **b** Quantitation of Dna2 or Dna2^6K^ protein levels from the experiments such as shown in **a**. The values are expressed relative to Dna2^6K^ levels at S-G2. Averages shown, *n* = 3; whiskers, s.e.m. **c** Real-time PCR was used to obtain cycle threshold levels (relative to Actin, ΔCt) to quantitate DNA2 and RAD27 mRNA levels from *DNA2* and *dna2*^*6K*^ cells (JKM139 background). Averages shown, *n* = 3; whiskers, s.e.m. **d** Comparison of Dna2 and Dna2^6K^ levels in whole cell (20 μg) and nuclear extracts (3.8 μg) (JKM139 background) by western blotting. Asterisk indicates a putative Dna2 truncation product. **e** Quantitative analysis of Dna2-YFP or Dna2^6K^-YFP foci. Cells (yLK354 and yLK388, W303 background) were treated with methyl-methanesulfonate (MMS, 0.015% for 3 h) and subjected to microscopy analysis. The fraction of cells showing Yellow Fluorescent Protein foci is shown. Average and SEM of three independent experiments are shown (*n* > 160 cells per strain). Statistical significance relative to the wild type was determined by Fisher’s exact test from the total numbers of cells with or without foci from all experiments

We next set out to test whether sumoylation controls the stability of the Dna2 protein. To this point, we synchronized wild-type *DNA2* and *dna2*^*6K*^ cells by α-factor in G1, and monitored total Dna2 protein levels upon release throughout the cell cycle (Fig. 4a, b). Notably, we observed that the Dna2^6K^ protein was ~2–3-fold more abundant in every phase of the cell cycle tested, as well as in asynchronous cells compared to its wild type counterpart. Sumoylation, however, did not affect the relative protein distribution throughout the cell cycle. Both wild-type and Dna2^6K^ proteins were most abundant in late S-G2 phase of the cell cycle, and least abundant in G1 (Fig. 4a, b). The DNA2 messenger RNA (mRNA) levels from *DNA2* and *dna2*^*6K*^ cells were identical, as anticipated (Fig. 4c), in agreement with the model that sumoylation reduces Dna2 protein stability through a post-translational mechanism. Previously, it has been reported that the non-phosphorylatable Dna2 S17A and S237A variants showed impaired nuclear localization^25^. To test whether the non-sumoylatable Dna2^6K^ translocates into the nucleus, we prepared nuclear extracts and compared the levels of wild-type Dna2 and Dna2^6K^ variants. As shown in Fig. 4d, Dna2^6K^ was more abundant only in total but not in nuclear extracts. The nuclear levels of Dna2^6K^ were comparable, or even slightly lower than that of wild-type Dna2 (Fig. 4d). This showed that the Dna2^6K^ variant is less efficiently imported into the nucleus than the wild-type protein. We also monitored the YFP-tagged Dna2/Dna2^6K^ by microscopy. While Dna2 shows little detectable staining or foci without DNA damage, we observed that Dna2^6K^ showed reduced recruitment to DNA damage sites compared to the wild-type protein (Fig. 4e). In summary, although the Dna2^6K^ mutant shows elevated protein levels in total cell extracts, the variant is not more abundant than wild-type Dna2 in the nucleus and shows reduced recruitment to sites of DNA damage.

### Non-sumoylatable Dna2 impairs DNA end resection

**Fig. 5:**
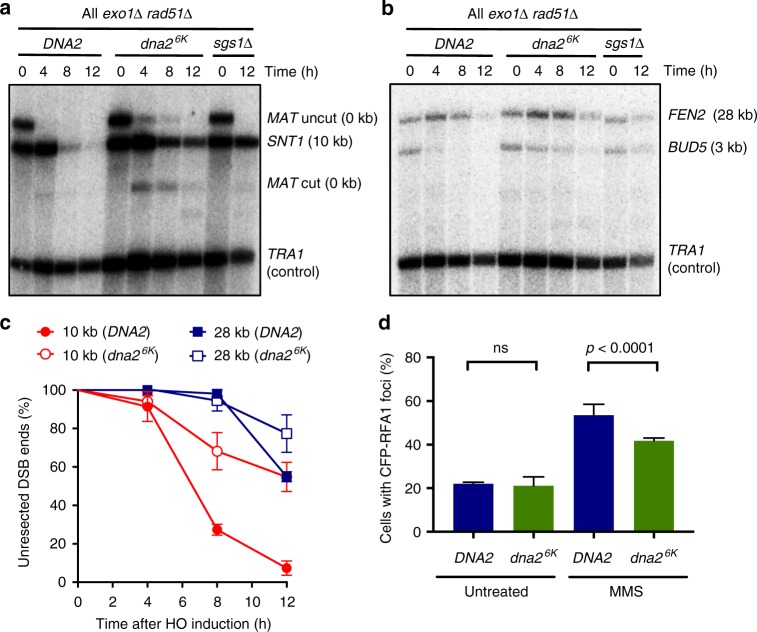
Non-sumoylatable *dna2*^*6K*^ cells are impaired in resection of HO-induced DNA breaks. **a** DNA at the *MAT* locus was cleaved upon induction of the HO endonuclease with galactose. DNA cleavage and end resection at the indicated time and distance (*MAT* locus, 0 kb and *SNT1*, 10 kb) was estimated by southern blotting in DNA2, *dna2*^*6K*^, or *sgs1*Δ cells (JKM139 background). All strains contained *exo1*Δ and *rad51*Δ mutations. *TRA1* was used as a loading control. **b** Experiment as in **a**, but measuring resection at *BUD5* (3 kb) and *FEN2* (28 kb) loci. **c** Quantitation of DNA end resection at indicated distances from the DSB from experiments such as shown in **a** and **b**. Averages shown, *n* = 3; error bars, s.e.m. **d** Quantitative analysis of Rfa1-CFP foci in *DNA2* or *dna2*^*6K*^ cells (W303 background). Yeast strains (yLK414, and yLK415) were grown under normal conditions or treated with methyl-methanesulfonate (MMS, 0.015% for 3 h) and subjected to microscopy analysis. The fraction of cells showing CFP-Rfa1 (Cyan Fluorescent Protein fused to the replication protein A subunit 1) foci is shown. Average and SEM of three independent experiments are shown (*n* > 160 cells per strain). Significance relative to wild type was determined by Fisher’s exact test from the total numbers of cells with or without foci from all experiments

Dna2 catalyzes long-range DNA end resection together with Sgs1 in a pathway that may act redundantly with the Exo1 nuclease^19^. To establish whether misregulated Dna2 affects resection, we analyzed DNA end processing in *DNA2* and *dna2*^*6K*^ cells in an *exo1*Δ background, which is needed to highlight defects in the Dna2 pathway^19^. To this point, we used an established system that measures DNA end resection by southern blotting^19^. DNA is cleaved at the *MAT* locus by HO endonuclease, the expression of which is induced by the addition of galactose^43^. Resected DNA, which becomes single-stranded, cannot be digested by a restriction endonuclease, leading to a loss of specific southern blot signals corresponding to locations at various distances from the DSB^19^. At all positions tested, we observed reduced DNA end resection in *dna2*^*6K*^ compared to *DNA2* cells (Fig. 5a–c). This was further confirmed by monitoring the levels of CFP-RPA foci as a read out of ssDNA accumulation. Wild-type cells treated with MMS showed higher accumulation of RPA foci in contrast to Dna2^6K^ (Fig. 5d). The impaired resection in *dna2*^*6K*^ cells is in agreement with the reduced nuclear localization and recruitment of the Dna2^6K^ variant.

### Non-sumoylatable Dna2 impedes growth rate

**Fig. 6:**
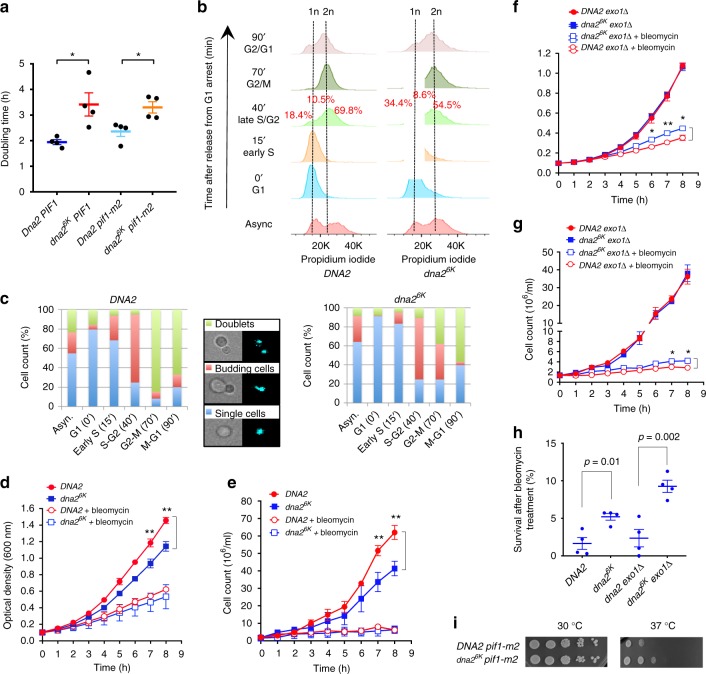
Non-sumoylatable Dna2 is toxic under normal growth conditions but beneficial upon stress. **a** Doubling times calculated from cell growth monitored by optical density measurements of wild-type *DNA2* and *dna2*^*6K*^ cells in wild-type *PIF1* or *pif1-m2* cells (JKM139 background), as indicated. Averages shown, *n* = 4; whiskers, s.e.m.; *p*-values were obtained with two-tailed *t*-test. **b** FACS analysis of cell cycle progression of *DNA2* and *dna2*^*6K*^ strains (JKM139 background). Cells were synchronized in G1 by α-factor and released for indicated times. 1n and 2n DNA content is indicated by vertical lines. **c** Cell cycle progression analysis of *DNA2* and *dna2*^*6K*^ cells (JKM139 background) synchronized as in **b**, but monitored by light or fluorescence (DAPI) microscopy. Quantitated were single cells (no visible bud), budding cells (with incomplete nuclear division) or doublet cells (complete nuclear division but cells still physically connected). **d** Growth of *DNA2* and *dna2*^*6K*^ strains (JKM139 background) without or with bleomycin (0.1 μg/ml), monitored by optical density measurements. Averages shown, *n* = 3; error bars, s.e.m.; **(*p* < 0.01, two-tailed *t*-test). **e** Growth of *DNA2* and *dna2*^*6K*^ strains (JKM139 background) without or with bleomycin (0.1 μg/ml), monitored by cell counts. Averages shown, *n* = 3; error bars, s.e.m.; **(*p* < 0.01, two-tailed *t*-test). **f** Growth of *DNA2 exo1*Δ and *dna2*^*6K*^ *exo1*Δ strains (JKM139 background) without or with bleomycin (0.1 μg/ml), monitored by optical density measurements. Averages shown, *n* = 4; error bars, s.e.m.; *(*p* < 0.05); **(*p* < 0.01) (two-tailed *t*-test). **g** Growth of *DNA2 exo1*Δ and *dna2*^*6K*^ *exo1*Δ strains (JKM139 background) without or with bleomycin (0.1 μg/ml), monitored by cell counts. Averages shown, *n* = 2; whiskers, range. **h** Cell (JKM139 background) survival, scored by colony-forming capacity, of indicated strains after treatment with bleomycin (0.1 μg/ml) for 8 h. Averages shown, *n* = 4; whiskers, s.e.m.; *p*-values were obtained with two-tailed *t*-test. **i** Temperature sensitivity of *DNA2* and *dna2*^*6K*^ strains (JKM139 background) in the *pif1-m2* background. Plates were incubated at 30 °C for 2 days or 37 °C for 3 days. Representative experiments are shown

To further describe the cellular phenotypes arising from impaired Dna2 sumoylation, we compared the doubling time of *DNA2* and *dna2*^*6K*^ strains, as determined by monitoring optical density of the cultures during exponential growth (Fig. 6a). We observed that the *dna2*^*6K*^ strain showed a significantly increased doubling time (lower growth rate) compared to wild-type cells (Fig. 6a). The inactivation of the Pif1 helicase was shown to bypass the requirement for Dna2 in Okazaki fragment processing during lagging strand DNA replication^44^. To determine whether DNA repair or replication functions of Dna2 are affected by preventing Dna2 sumoylation, we used the *pif1-m2* mutant, which affects nuclear, but not mitochondrial functions of Pif1^45^. The reduced growth rate was also apparent in the *pif1-m2* background (Fig. 6a and Supplementary Fig. 3a). This did not exclude a potential function in DNA replication, but suggested that impaired Dna2 sumoylation also affects pathways other than Okazaki fragment processing. Furthermore, in *siz2*Δ cells, where Dna2 sumoylation is reduced (Fig. 1b), the *dna2*^*6K*^ mutation did not affect the growth rate (Supplementary Fig. 3b). We note that the *siz2*Δ mutation brought about reduced growth rate in both *dna2*^*6K*^ and *DNA2* backgrounds, likely due to a global defect in sumoylation (Supplementary Fig. 3b). Nevertheless, the result suggests that the growth rate reduction is likely linked to the reduced sumoylation of Dna2^6K^. Strains bearing *dna2* variants with less than six mutated lysines did not show an obvious delay in doubling rate, although we note that we were not able to test the K21 mutant alone (Supplementary Fig. 3a). This suggests that Dna2 is either sumoylated alternatively at all six lysine residues, or the inability to sumoylate Dna2 at one position shifts the preference towards another residue in the vicinity.

Analysis of cell cycle distribution by fluorescence-activated cell sorting (FACS) showed  that a higher proportion of asynchronous *dna2*^*6K*^ cells accumulated in the G2 phase of the cell cycle (Fig. 6b and Supplementary Fig. 3c). Synchronization experiments then revealed that the inability to sumoylate Dna2 slows down the capacity of the yeast cells to progress through S phase, independently of *PIF1* status (Fig. 6b and Supplementary Fig. 3d, e). This was separately confirmed by observing cellular and nuclear morphology by microscopy during synchronization. The appearance of budding cells upon release from G1 was similar in both *dna2*^*6K*^ and *DNA2* cells (Fig. 6c). However, at 70 min post-release, ∼85% of wild-type cells completed nuclear division and were in the process of cytokinesis. In contrast, at the same time point, only 38% of *dna2*^*6K*^ cells reached this stage (Fig. 6c). Altogether, our data show that strains with non-sumoylatable Dna2 exhibit delayed progression through S phase, which results in impaired growth rate. The reduced growth of *dna2*^*6K*^ cells was reminiscent of a growth arrest observed upon Dna2 overexpression shown previously^46^. Likewise, *dna2*^*Δ405N*^ cells lacking the N-terminal domain of Dna2, which hyperactivates the Dna2 nuclease, also grow poorly^41^.

### Non-sumoylatable Dna2 is beneficial upon stress

Next, we explored the role of Dna2 sumoylation upon the induction of DNA damage by the radiomimetic drug bleomycin, which causes DNA breaks. The *DNA2* and *dna2*^*6K*^ cells were grown in rich medium without or with bleomycin, and cell growth was monitored by optical density measurements and cell counting. As above, we observed a significantly reduced growth rate of *dna2*^*6K*^ cells compared to wild-type cells without induced DNA damage (Fig. 6d, e). With bleomycin, in contrast, the growth of *DNA2* and *dna2*^*6K*^ cells was very similar (Fig. 6d, e). Likewise, in *exo1*Δ background, without DNA damage, the growth of *DNA2* and *dna2*^*6K*^ cells was indistinguishable (Fig. 6f, g). Upon bleomycin treatment, however, *dna2*^*6K*^ *exo1*Δ cells in contrast grew better than *DNA2 exo1*Δ cells, showing that *dna2*^*6K*^ mutation provides growth advantage under conditions of genotoxic stress in the *exo1*Δ background (Fig. 6f, g). The *dna2*^*6K*^ mutation was similarly beneficial in cell survival assays upon bleomycin treatment, in both *EXO1* and *exo1*Δ backgrounds (Fig. 6h). Finally, *dna2*^*6K*^ cells were less temperature sensitive than *DNA2* in the *pif1-m2* background (Fig. 6i). The *dna2*^*6K*^ mutation did not result in a detectable change in checkpoint activation, as scored by Rad53 phosphorylation, without or with bleomycin treatment (Supplementary Fig. 4a). Likewise, we did not detect altered amount of DNA double-strand breaks in *dna2*^*6K*^ cells compared to wild type upon DNA damage (Supplementary Fig. 4b). In summary, we show that in contrast to unperturbed conditions where *dna2*^*6K*^ cells are impaired, this mutation can be beneficial upon genotoxic stress. This collectively indicates that Dna2 levels must be properly balanced to optimally promote genome stability.

## Discussion

Dna2 is a nuclease with multiple roles in DNA metabolism, which remain to be fully defined^12,15,19^. In order to support its diverse functions, the activity and stability of Dna2 must likely be tightly regulated to allow efficient DNA processing, but not at the cost of unscheduled DNA degradation. The results presented here reveal that yeast Dna2 is post-translationally modified by sumoylation. We found that sumoylation targets multiple residues located within the N-terminal domain of Dna2, which has a known regulatory function. Previously, it was described that the N-terminal domain of Dna2 is phosphorylated by CDK, which facilitates nuclear import and recruitment to DNA, which in turn promotes DNA end resection^25^. Sumoylation, as revealed here, likely regulates the Dna2 functions by several mechanisms.

In total cell extracts, we found that the non-sumoylatable Dna2 mutant (denoted Dna2^6K^) was ∼2–3-fold more abundant than wild-type Dna2. Sumoylation often promotes proteolysis, which is mediated by SUMO-targeted ubiquitin ligases in both yeast and humans^6,47^. For example, human FEN1 and the FANCI-FANCD2 complex are sumoylated, which precedes their ubiquitin-mediated proteasomal degradation^48,49^. Sumoylation also promotes the proteasomal degradation of human EXO1, while yeast Exo1 is likewise sumoylated^30^. Jentsch and colleagues identified a sumoylation wave that occurs in response to DSBs that requires extensive resection^9^. As several resection pathways are regulated by sumoylation^27,30^, modification of resection proteins might thus represent a feedback loop. Importantly, while Dna2^6K^ levels were elevated in total cell extracts, we found that the Dna2^6K^ variant was no more abundant than the wild-type protein in nuclear extracts, and showed a defect in recruitment to DNA damage sites. Therefore, similarly as the non-phosphorylatable Dna2 variant^25^, we found that Dna2^6K^ was partially impaired in nuclear import and recruitment. As a result, *dna2*^*6K*^ cells showed a modest defect in DNA end resection. The mechanism how phosphorylation and sumoylation regulate these transactions, as well as whether there is a crosstalk between the two processes, remains to be defined.

Furthermore, using a reconstituted system, we show that sumoylation specifically reduces the nuclease, but not DNA binding, helicase or ATPase activities of recombinant Dna2. Seo and colleagues observed that the N-terminal domain binds a Dna2 region located further downstream between the nuclease and the helicase domains, and proposed that the N-terminus of Dna2 might fold over the nuclease domain to limit its activity^41^. Consequently, Dna2^Δ405^, a variant lacking the N-terminal domain, displays higher levels of nuclease activity compared to the full-length polypeptide in vitro, and the *dna2*^Δ*405*^ mutation impairs cell growth^41^. Our observations support the role of the N-terminal domain in Dna2 regulation as its sumoylation limits the Dna2 nuclease activity. We note that the fraction of sumoylated Dna2 protein at any time in cells is very small, so inhibition of the Dna2 nuclease by sumoylation cannot affect the activity of most Dna2 molecules in the protein pool. However, it may be relevant if Dna2 is preferentially modified “on site”, i.e., when bound to a particular DNA substrate or a protein partner^7^.

What are the other consequences of abrogated Dna2 sumoylation? We observed that the non-sumoylatable *dna2*^*6K*^ strain displayed a notably decreased growth rate characterized by defects in timely progression through the S and G2 phases of the cell cycle. Balanced levels of the Dna2 protein and activity are required for optimal growth conditions. Lack of Dna2 is lethal, while higher than physiological Dna2 levels are toxic as well. Overexpression of Dna2 was shown to lead to growth arrest^46^. Also, *dna2*^Δ*405*^ cells, lacking the Dna2 N-terminal domain, displayed reduced growth rate, while the recombinant Dna2^Δ405^ mutant is more active as a nuclease than the full-length protein in vitro^41^. In contrast to normal growth conditions, we found that non-sumoylatable Dna2 can be beneficial upon high levels of genotoxic stress. Specifically, we observed that *dna2*^*6K*^ cells did not show reduced growth rate compared to wild-type cells in the presence of the radiomimetic drug bleomycin. Instead, the *dna2*^*6K*^ mutation even provided growth advantage in the *exo1*Δ background, or in assays where cell survival was monitored upon prolonged exposure to bleomycin. As Dna2 is involved in multiple DNA metabolic pathways, some of which are not yet understood, linking the phenotypes of the *dna2*^*6K*^ cells with a particular function is challenging. To determine whether sumoylation controls DNA replication or repair functions of Dna2, we analyzed the phenotypes of *dna2*^*6K*^ cells in wild-type *PIF1* or *pif1-m2* background, which abrogates the necessity for Dna2 in Okazaki fragment processing^44^. The phenotypes of *dna2*^*6K*^ cells were not affected by the *pif1-m2* mutation. This did not exclude a potential effect of Dna2 sumoylation on Okazaki fragment processing, but indicated that sumoylation also controls Dna2 functions separate from lagging strand DNA replication. The precise mechanism however remains to be defined.

Finally, we found that Dna2 protein levels vary during the cell cycle. Dna2 amounts were lowest in G1 and early S, increased throughout S to peak in the G2/M phase of the cell cycle. This variation was not affected by sumoylation, as *dna2*^*6K*^ cells showed a similar variation. The Dna2 expression profile differs significantly from that of FEN1/Rad27, which is mostly expressed in early S phase^48^. Yeast Dna2 and Fen1 were proposed to function together in the processing of long DNA flaps during Okazaki fragment maturation^12,50^. The different expression profile of Dna2 is in agreement with our previous data that Dna2 may also function in long DNA flap processing separately from FEN1^51^, or reflect an Okazaki fragment processing independent function of Dna2 later in the cell cycle. To this point, yeast *dna2*Δ cells arrest in G2-M and human cells with depleted DNA2 arrest in late S-G2, while yeast *rad27*Δ (FEN1-deficient) cells accumulate already in S phase^52–54^. This supports the notion that FEN1/Rad27 and Dna2 act in only partially overlapping pathways^51^.

The N-terminal regulatory domain of Dna2 is not present in the human homolog, suggesting that the mechanism of Dna2/DNA2 regulation may not be directly conserved. However, increased expression of human DNA2 was observed in multiple cancers, which was proposed to counteract replication stress^55,56^. As elevated replication stress is a hallmark of cancer cells, increased DNA2 expression might help tumor cells overcome this barrier. Together, the data presented here identify mechanisms that help regulate Dna2 function, which needs to be balanced to promote genome stability.

## Methods

### Recombinant proteins

Wild-type Dna2, Dna2 E675A, and Dna2 K1080E were expressed as fusions with N-terminal FLAG tag and C-terminal His tag. The protein was expressed from a galactose inducible promoter in yeast, and purified by affinity chromatography using Ni-NTA agarose (Qiagen) and M2 anti-FLAG affinity gel (Sigma)^39^. Genes coding for the Dna2^Δ405N^ variants (wt, E675A and K1080E), as well as for full-length Dna2^6K^ (six lysine residues replaced by alanines) were fused with N-terminal FLAG tag and a C-terminal His tag, cloned into a pYes2 vector (ThermoFisher) and expressed and purified as the full-length proteins. Yeast RPA was expressed in *Escherichia coli* and purified using HiTrap Blue, HiTrap Desalting and HiTrap Q chromatography columns (GE Healthcare)^57^. The *S. cerevisiae* SUMO machinery proteins, including GST-Aos1/Uba2, His-Ubc9, His-FLAG-Smt3-KR (all lysines substituted by arginines, denoted as Smt3 in the text), His-Siz1 (1–465) and His-Siz2 were expressed in *E. coli*, and purified by affinity and ion exchange chromatography^58,59^. Yeast nuclear extracts were prepared by a standard procedure^60^.

### In vitro sumoylation assays and mass spectrometry analysis

In vitro sumoylation assays were performed in 20 μl reactions by incubating recombinant Dna2 (350 ng) with GST-Aos1/Uba2 (200 ng), His-Ubc9 (160 ng), His-Siz1 (60 ng) and His-FLAG-Smt3-KR (800 ng) in a buffer containing 50 mM Tris-HCl (pH 7.5), 5 mM magnesium chloride and 1 mM ATP for 40 min at 30 °C^61^. Proteins were then directly used for biochemical assays (upon appropriate dilution) or were separated by electrophoresis in polyacrylamide gels and visualized by silver staining using standard procedures. The mass spectrometry analysis of sumoylated Dna2 was carried out by Dorothea Anrather and Gustav Ammerer in Max. F. Perutz Laboratories, Vienna, Austria.

### DNA substrates

Oligonucleotides X12-3 and X12-4SC were used for the preparation of the 5′-tailed ssDNA substrate with 19 nt-long ssDNA overhang, and oligonucleotides PC292 and X12-4SC for the preparation of the 30 nt-long 5′-tailed substrate^39,62^. The oligonucleotides used for the nuclease and helicase assays were ^32^P-labeled at the 5′ terminus with [γ-^32^P] ATP (Perkin Elmer) and T4 polynucleotide kinase (New England Biolabs) according to the manufacturer’s instructions. Unincorporated nucleotides were removed using MicroSpin G25 columns (GE Healthcare) before annealing of the substrates. The randomly labeled 2200 nt-long substrate was prepared by random incorporation of [α-^32^P] dATP (Perkin Elmer) into a PCR product, using LigFor and LigRev primers and yeast genomic DNA (yWH436 strain) as a template^20^. The sequences of oligonucleotides are listed in Supplementary Table 1.

### Nuclease, helicase, and ATPase assays

The experiments were performed in 15 μl volume in a reaction buffer containing 25 mM Tris-acetate (pH 7.5), 2 mM magnesium acetate, 1 mM ATP, 1 mM dithiothreitol (DTT), 0.1 mg/ml bovine serum albumin (BSA, New England Biolabs), 1 mM phosphoenolpyruvate, 16 units/ml pyruvate kinase (Sigma), 1 nM DNA substrate (in molecules), 16.8 nM RPA in case of 5′-tailed oligonucleotide-based substrates and 350 nM RPA in case of 2200 nt-long substrate. The reactions were assembled on ice and Dna2 variants were added, as indicated. The reactions with oligonucleotide-based substrates were incubated for 30 min at 30 °C^62^. Reactions with the 2200 nt-long substrate were incubated at 30 °C and aliquots were taken at indicated time points. Reactions were stopped by adding an equal volume of formamide dye (95% formamide, 20 mM ethylenediaminetetraacetic acid [EDTA], 0.03% bromophenol blue), samples were heated at 95 °C for 4 min and separated on 20% denaturing polyacrylamide gels (ratio acrylamide:bisacrylamide 19:1, Biorad). After fixing in a solution containing 40% methanol, 10% acetic acid and 5% glycerol for 30 min, the gels were dried on DE81 chromatography paper (Whatman) and exposed to storage phosphor screens (GE Healthcare)^63^. The screens were scanned by Typhoon phosphor imager (GE Healthcare), and data were quantitated using ImageQuant software. ATPase assay was based on a reaction in which the regeneration of hydrolyzed ATP is coupled to the oxidation of NADH, which can be monitored spectrophotometrically in real-time. The reaction buffer contained the indicated concentrations of Dna2 (or Sumo-Dna2) and 1 μM (nucleotides) nucleic acid cofactors, 1 mM ATP, 25 mM Tris-acetate (pH 7.5), 1 mM magnesium acetate, 0.1 mM DTT, 1 mM phosphoenolpyruvate, 25 units/ml pyruvate kinase (Sigma), 25 units/ml l-lactate dehydrogenase (Sigma), and 200 μM NADH (Sigma)^62,64^.

### DNA-binding assays

The experiments were performed in a 15 μl volume in a binding buffer containing 25 mM Tris-acetate (pH 7.5), 1 mM EDTA, 1 mM DTT, 0.1 mg/ml BSA and 1 nM (in molecules) ^32^P-labeled DNA substrate. After the addition of the sumoylated or mock-treated Dna2 E675A, the reactions were incubated at 30 °C for 30 min, mixed with 4 μl loading dye (50% glycerol, 0.03% bromophenol blue) and separated on 6% polyacrylamide gels. The gels were then dried and processed as noted above.

### Yeast strains and plasmids

The experiments shown in Fig. 1 and Supplementary Fig. 1 were performed using *S. cerevisiae* FF18733 strain (F. Fabre). The *DNA2* gene was C-terminally tagged with 9-MYC tag using pYM18 plasmid^65^. Plasmid variants used for His-Smt3 pulldown experiments were YEp181-CUP1-His-SMT3 and the corresponding empty vector (a kind gift from H. Ulrich, IMB, Mainz). All other experiments were performed using JKM139-derived and yWH436 strains^19^. The *dna2*^*6K*^ mutant (and variants) with lysines replaced by alanines were constructed by allele replacement strategy using pRS306 plasmid that included selection on minimal media lacking uracil and counterselection with 5-fluoroorotic acid^42^. Gene deletions were conducted as follows: Δ*dna2::URA3* (template for PCR-based deletion cassette pUG72)*,* Δ*siz1*::*URA3* (pUG72), Δ*siz2::hphNT1* (pFA6), Δ*exo1*-*hphNT1* (pFA6), Δ*rad51*-*KanMX* (pUG6)^65^. The imaging of CFP-Rfa1 or YFP-Dna2  was carried out with strains derived from W303. For full strain list see Supplementary Table 2. Primer sequences are available upon request.

### Western blot analysis

Cell extracts were prepared by lysing the cells (from 2 ml culture for input and 50 ml culture for Ni-NTA pulldowns) with 1.85 M sodium hydroxide and 7.4% (v/v) beta-mercaptoethanol followed by protein precipitation with Trichloroacetic acid (10% [v/v] final, PanReac Applichem). Samples were centrifuged (3000 × *g* for 5 min), the protein pellets were solubilized and denatured in the denaturing buffer (8 M urea, 200 mM Tris-HCl (pH 6.8), 1 mM EDTA, 5% sodium dodecyl sulfate (SDS), 1.5% DTT, 0.03% bromophenol blue) for 10 min at 60 °C before electrophoresis^32^. Protein electrophoresis and western blotting were performed according to standard procedures. Following antibodies were used: anti-c-Myc-tag mAb (1:1000, A00704, GenScript), anti-Smt3 (1:1000, ab14405, Abcam), anti-His-tag mAb (1:2500, A00186, GenScript), anti-Rad53 (1:1000, yC-19, sc-6749, Santa Cruz Biotechnology), anti-α-Tubulin (1:1000, YOL1/34, sc-53030, Santa Cruz Biotechnology). Uncropped blots are presented in Supplementary Fig. 5.

### Detection of Smt3-Dna2 conjugates

Yeast cells transformed with Yep181-CUP1-His-SMT3 or YEplac181 were grown exponentially until OD_600_ = 1 in minimal medium. Smt3 expression was induced with copper sulfate (100 μM final), which was also used to treat samples transformed with an empty vector (no His-Smt3) as a control. Cells were lysed under denaturing conditions as described above. Proteins were precipitated and purified by Ni-NTA affinity chromatography^32^. Sumoylation of Dna2 was analyzed by western blotting. Genotoxic treatments were conducted for 1.5 h before harvesting using the following drugs (final concentrations, unless indicated otherwise): methyl-methanesulfonate (MMS, 0.03%, Sigma-Aldrich), bleomycin (5 μg/ml, Bleocin, Merck Millipore), etoposide (295 μg/ml, Sigma-Aldrich).

### Cell synchronization and flow cytometry

Cells were grown until OD_600_ = 0.35 and synchronized by the addition of α-factor (4 μg/ml, Primmbiotech) in YPD medium (2% bacto peptone, 1% yeast extract, 2% glucose) or minimal medium for 2 h. Cells were released into S-phase by treatment with Pronase (20 mg/ml, Sigma-Aldrich). Samples were collected for flow cytometry and Ni-NTA pulldowns at indicated time points. For flow cytometry, cells were fixed in 70% ethanol and 250 mM Tris-HCl (pH 7.5) at 4 °C overnight, treated with RNase A (1 mg/ml, Roche) at 37 °C overnight, washed and resuspended in 50 mM Tris-HCl (pH 7.5), 200 mM NaCl, 80 mM MgCl_2_ and propidium iodide (50 μg/ml, Sigma-Aldrich). After brief sonication in 50 mM Tris-HCl (pH 7.5), the DNA content was measured using CyAn ADP 9 flow cytometer (Beckman Coulter) operated with Summit software and the data was analyzed with FlowJo software (TreeStar).

### MG132 treatment

Yeast strains were grown overnight in minimal medium containing 0.1% proline and diluted into the same medium containing additionally 0.003% SDS to permeabilize the cells^66^. After 1 h, α-factor (3 μg/ml, Primmbiotech) and 75 μM MG132 (Sigma) or an equivalent volume of DMSO were added to the cultures. Samples for flow cytometry and western blot analysis were collected at indicated time points.

### Pulsed-field gel electrophoresis

Pulsed-field gel electrophoresis was performed at 14 °C in 0.9% (w/v) Pulsed Field Certified Agarose (Biorad) containing TBE buffer (89 mM Tris-borate, 2 mM EDTA) in a CHEF DR III apparatus (9 h, 120°, 5.5 V/cm, 30–18 s switch time; 6 h, 117°, 4.5 V/cm, 18–9 s switch time; 6 h, 112°, 4 V/cm, 9–5 s switch time; Biorad)^67^. The agarose gel was stained with ethidium bromide and imaged on an Alpha Innotech Imager.

### Growth curves and DNA damage sensitivity assays

Indicated yeast strains were grown overnight in YPD medium (2% bacto peptone, 1% yeast extract, 2% glucose). Next morning, the cells were diluted into fresh YPD medium with or without bleomycin (0.1 μg/ml) at OD_600_ = 0.1. At indicated time points, growth was monitored by optical density measurements at 600 nm (OD_600_) or by cell counts. To monitor cell survival, the cells were treated with bleomycin (0.1 μg/ml for 8 h) and plated at various dilution on solid YPD medium without the drug. After 2–3 days, the colonies were counted, and expressed as a fraction of survivors from treated vs. non-treated cultures. To monitor temperature sensitivity, various dilutions of the indicated strains were plated on solid YPD medium. The plates were then incubated for 2 days at 30 °C or 3 days at 37 °C.

### Quantitative reverse transcription PCR (qRT-PCR)

*Dna2* and *dna2*^*6K*^ cells were lysed in three volumes of Trizol (TRI REAGENT, TR118, Molecular Research Center) and RNA was isolated using RNA extraction kit (Zymo Research). RNA (500 ng) was reverse transcribed to complementary DNA (cDNA) using qScript cDNA SuperMix (QuantaBio) according to the manufacturer’s instructions. The qRT-PCR reaction was carried out by mixing corresponding cDNA (25 ng), forward and reverse primers with PerfeCTa SYBR Green Fastmix (QuantaBio) as mentioned in manufacturer’s guidelines. *RAD27* (orthologue of human FEN1) was used as a control. The primers used are listed in Supplementary Table 1. The RT-PCR was performed in ABI-7900 HT system (Applied Biosystems) and the mRNA levels are reported as ΔCt values relative to Actin.

### Fluorescence microscopy

Cells were grown shaking in liquid SC+Ade medium at 25 °C overnight. Next day the cultures were diluted in fresh SC+Ade medium to OD600 = 0.25, grown for 2–3 hours and processed for fluorescence microscopy^68^. CFP-Rfa1 or YFP-Dna2  foci were visualized on a DeltaVision Elite microscope (Applied Precision, Inc.) equipped with a 100 × objective lens. Images were acquired using softWoRx (Applied Precision, Inc.) software. Image analysis and fluorescence intensity quantification were done using Volocity software (Perkin Elmer).

### Nuclear staining with 4′,6-diamidino-2-phenylindole (DAPI)

Cells were grown in YPD medium (50 ml) to OD_600_ = 0.35, synchronized in G1 phase by adding 3 μg/ml α-factor for 1.5 h, released into S-phase upon 20 mg/ml Pronase treatment. Samples for DAPI staining and FACS were collected at indicated time points upon release. For DAPI staining, one volume of cells was fixed with 2 volumes of 100% ethanol for 1 h. Cells were pelleted (5000 × g for 1 min) and washed with PBS, followed by the addition of DAPI (Sigma-Aldrich, 1:2000). The images of stained cells were visualized and captured using Leica Sp5 confocal microscope (Leica microsystems).

### In vivo resection assay

Cells were grown overnight in YPD (2 ml, 2% bacto peptone, 1% yeast extract, 2% glucose), medium was then changed to YP-lactate (4 ml, 2% bacto peptone, 1% yeast extract, 2% sodium lactate) and grown until OD_600_ increased by >0.1. Next, cells were diluted into fresh YP-lactate medium (500 ml) for another 24 h. HO endonuclease was induced at OD_600_ = 0.5 by the addition of 2% galactose. Samples (100 ml) were collected at indicated time points. Sample “0 h” was collected immediately after the addition of galactose. Cells were washed once with water and then with 100% ethanol (5 ml). The pellet was air-dried, resuspended in 3 ml TE buffer pH 7.5, flash frozen and ground in a freezer mill (6875, SPEX SamplePrep). Chromosomal DNA isolation was carried out using standard phenol extraction procedure. Chromosomal DNA (1 μg) was digested with *Eco*RI-HF enzyme (New England BioLabs), separated on 0.8% agarose gel, transferred onto a nylon membrane (GE Healthcare) and hybridized with ^32^P-labeled DNA probes^19^. The labeling of the probes was carried out by random primed DNA labeling kit (Roche) according to manufacturer’s instructions. Primers used to prepare the ^32^P-labeled probes are listed in Supplementary Table 1. Membranes were exposed to storage phosphor screens (GE Healthcare) that were scanned by a Typhoon 9400 phosphor imager (GE Healthcare). Band intensities were normalized to *TRA1* control band and quantitated as percentage of normalized band intensity at 0 h using ImageQuant TL (GE Healthcare).

### Reporting summary

Further information on research design is available in the Nature Research Reporting Summary linked to this article.

## Supplementary information

Supplemental Information

Description of Supplementary Data

Supplementary Data 1

Reporting Summary

Peer Review File

## Data Availability

Source data are available as Supplementary Data 1. All other data are available from the corresponding author upon reasonable request.
