# Supplementary material for: Sumoylation regulates the stability and nuclease activity of Saccharomyces cerevisiae Dna2
Source: Commun Biol. 2019 May 8;2:174. doi: 10.1038/s42003-019-0428-0 (PMC6506525; doi:10.1038/s42003-019-0428-0)
Supplement: Supplementary file 1 — Supplemental Information [file 42003_2019_428_MOESM1_ESM.pdf]

Supplementary Figure 1

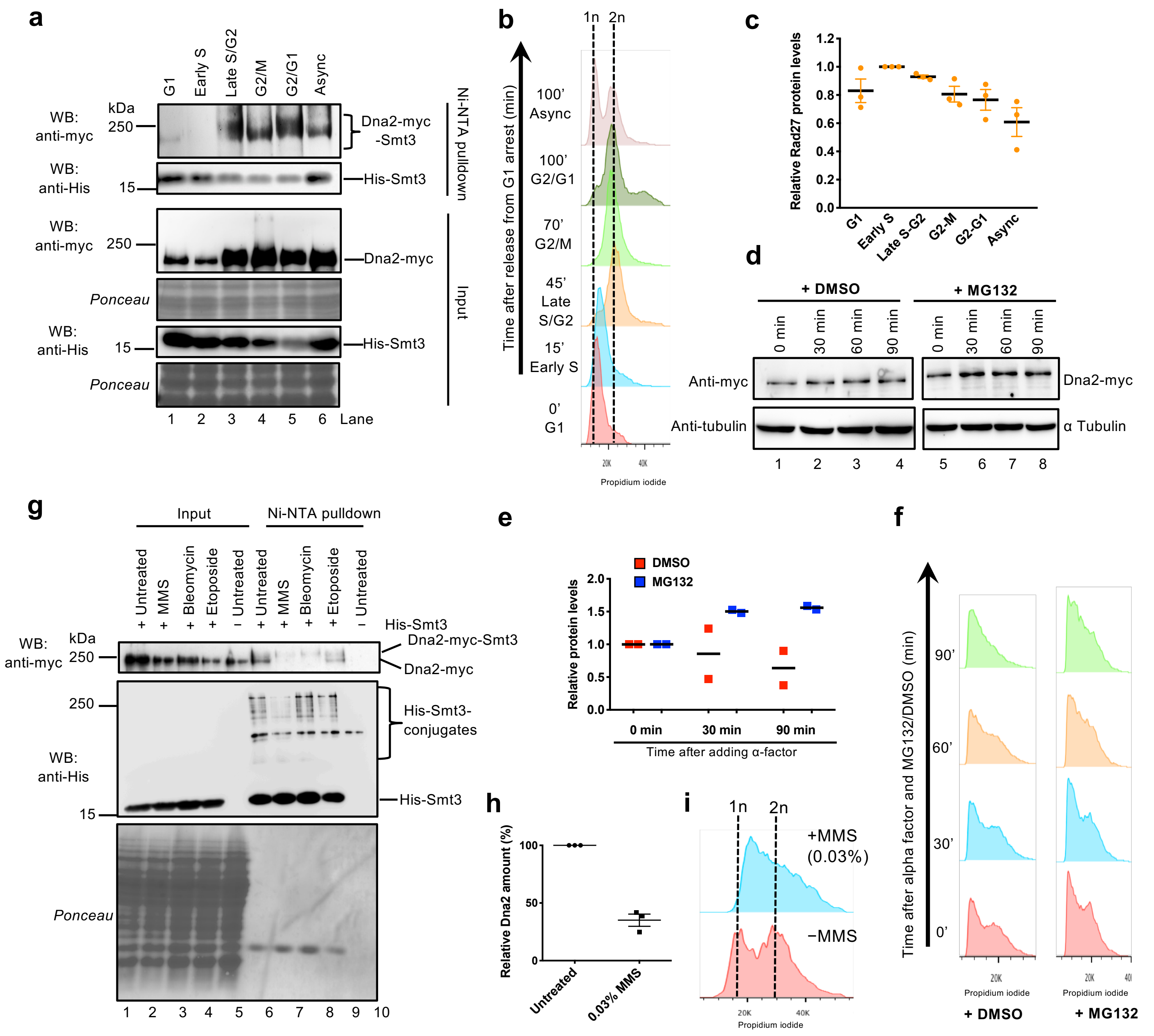

Supplementary Figure 1 Dna2 sumoylation and protein levels vary through cell cycle. (**a**) Sumoylation of Dna2 was monitored by Ni-NTA pulldown of His-Smt3 protein conjugates upon cell synchronization (FF18733 background) with α-factor. Representative western blot (WB) from three independent experiments is shown. Ponceau stained membrane sections shown serve as controls for equal loading. (**b**) Cell cycle progression of cells used in (a) was monitored by fluorescence-activated cell sorting, 1n and 2n DNA content is indicated by vertical lines. (**c**) Quantitation of Rad27 protein levels through cell cycle (JKM139 background). The values are expressed relative to the protein levels at early S phase. The experiment was performed with myc-tagged Rad27 upon synchronization with α-factor. Averages shown, n=3; error bars, s.e.m. (**d**) Representative western blot showing Dna2 protein levels upon MG132 treatment during synchronization with α-factor. Strains (FF18733 background) were grown in medium containing 0.1% proline and 0.003% SDS, then treated with 3 µg/ml α-factor and 75 µM MG132 (Sigma) or DMSO. Samples were taken after 0, 30, 60 and 90 min of treatment. (**e**) Quantitation of Dna2 levels from experiments such as shown in (d), relative to tubulin levels. The band intensity at time 0 min was set to 1. Averages shown, n=2. (**f**) Cell cycle progression analysis by fluorescence-activated cell sorting of samples from experiment in (d). (**g**) Ni-NTA pulldown of His-Smt3 protein conjugates from extracts of *S. cerevisiae* cells (FF18733 background) expressing myc-tagged Dna2. Cells were transformed with His-Smt3 plasmid or an empty vector as a negative control. Input and pulldown samples were analyzed by western blotting (WB) using anti-myc and anti-His antibodies. Cells were treated with MMS (0.03%), bleomycin (5 µg/ml) or etoposide (0.5 mM) for 1.5 h, as indicated. Representative western blots from three independent experiments are shown. (**h**) Quantitation of sumoylated Dna2 in extracts from control or MMS-treated cells (FF18733 background). The Dna2 band intensity from untreated cells was set to 100. Averages shown, n=3; error bars, s.e.m. (**i**) Cell cycle analysis of MMS treated (+MMS) and untreated (–MMS) cells from experiment such as shown in (h) by fluorescence-activated cell sorting. 1n and 2n DNA content is indicated by vertical lines.

Supplementary Figure 2

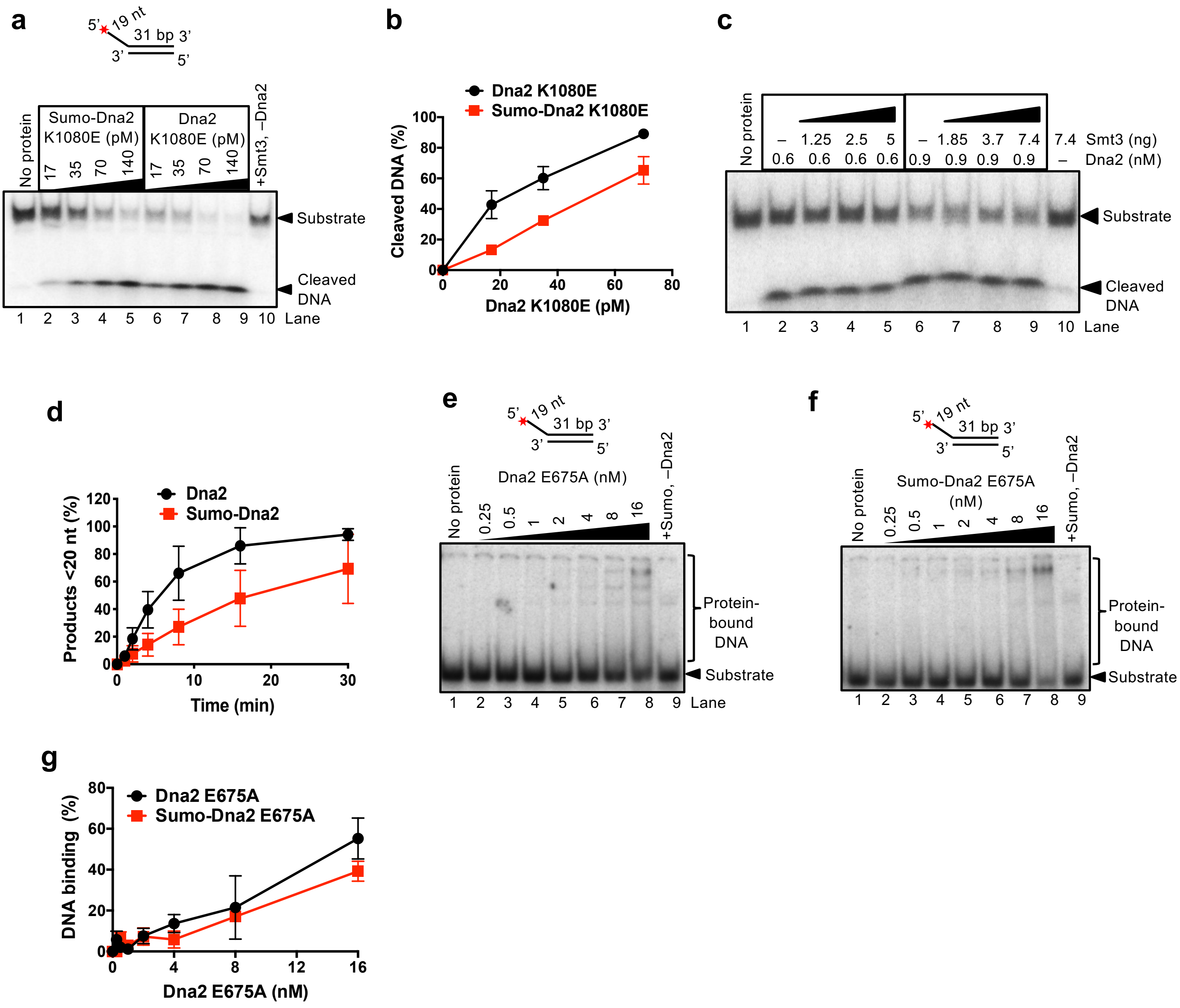

Supplementary Figure 2 *In vitro* sumoylated Dna2 exhibits decreased nuclease activity, while DNA binding is not affected by sumoylation. (**a**) Representative nuclease assays showing an experiment as in Fig. 2b, but with helicase-dead Dna2 K1080E. (**b**) Quantitation of experiments such as shown in (a). Averages shown, n=2; whiskers, range. (**c**) Representative nuclease assays showing the Dna2 nuclease activity in the presence of various amounts of the Smt3 protein. 2.5 ng Smt3 corresponds to the amount used in *in vitro* sumoylation assays. This and similar Smt3 concentrations do not affect the Dna2 nuclease. (**d**) Quantitation of experiments such as in Fig. 2g,h. Shown is the proportion of DNA fragments shorter than 20 nt in length, out of total DNA in each lane. Averages shown, n=2; whiskers, range. (**e**) and (**f**) Representative gels showing the binding of mock-sumoylated and sumoylated nuclease-dead Dna2 E675A to a 5'-tailed substrate. (**g**) Quantitation of DNA binding experiments such as shown in (e) and (f). Averages shown, n=3; error bars, s.e.m.

Supplementary Figure 3

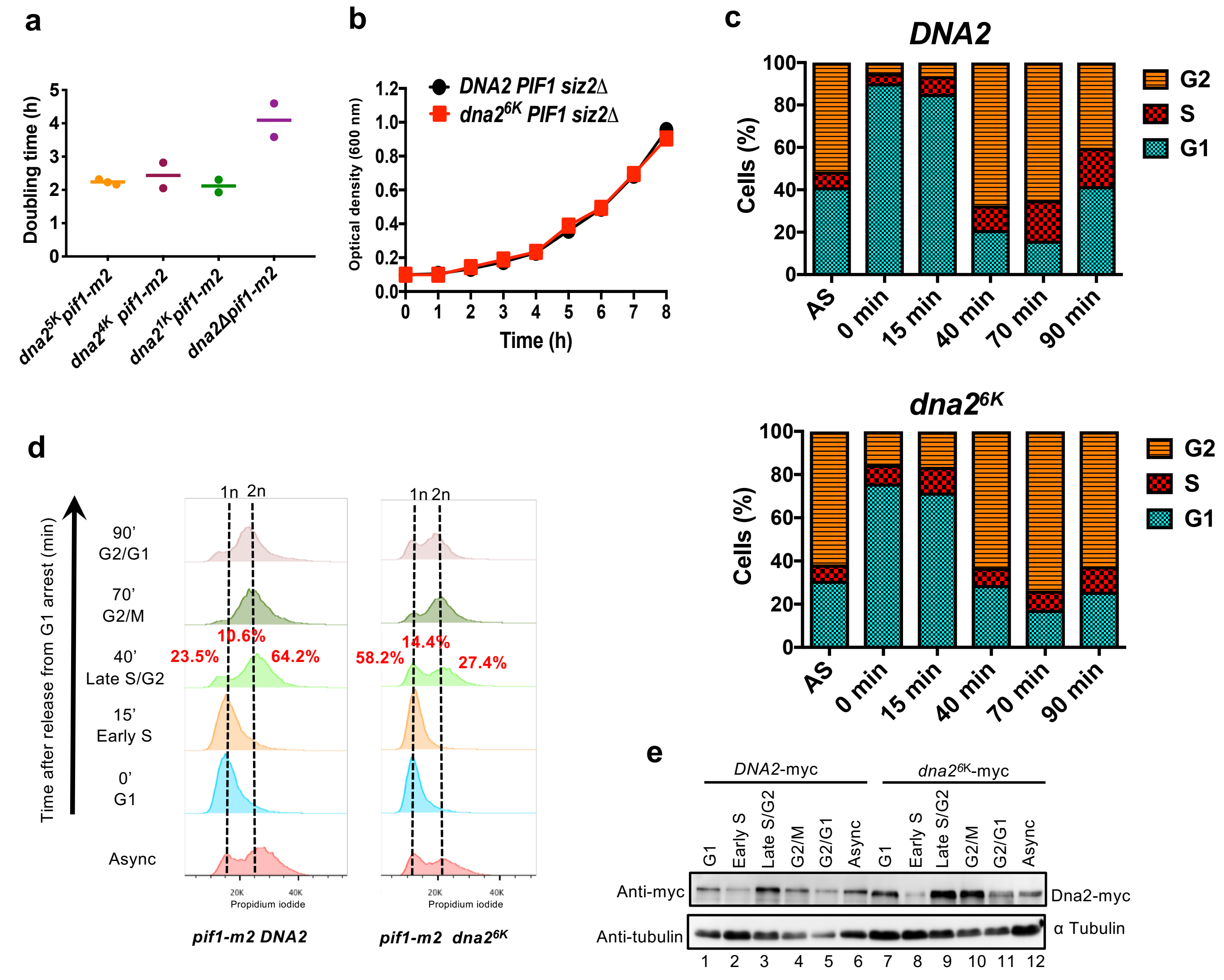

Supplementary Figure 3 Cells expressing Dna2^{6K} show slow cell cycle progression. (**a**) Doubling times calculated from growth monitored by optical density measurements of indicated strains (JKM139 background) in the *pif1-m2* background. Dna2^{5K} contains K33A, K60A, K93A, K103A, K247A, Dna2^{4K} contains K60A, K93A, K103A, K247A and Dna2^{1K} contains K247A. Averages shown, n=2; whiskers, range. (**b**) Growth of wild type *DNA2* or *dna2^{6K}* strains (JKM139 derived) in *siz2*Δ background, as estimated by optical density measurements. Averages shown, n=2; whiskers, range. (**c**) Analysis of cell cycle profiles from synchronization experiments such as shown in Fig. 6b. Shown is the proportion of cells in various stages of the cell cycle. Averages shown, n=2. AS, asynchronous cells. (**d**) Cell cycle progression of wild type and *dna2^{6K}* strains in *pif1-m2* (JKM139 background), which had been synchronized in G1 by α-factor and released into S phase (1n and 2n DNA content indicated). (**e**) Representative western blot analysis of wild type Dna2 and Dna2^{6K} expression levels at various stages of the cell cycle upon synchronization as shown in Fig. 6b. Western blot using anti-tubulin antibody was used as a loading control.

Supplementary Figure 4

a

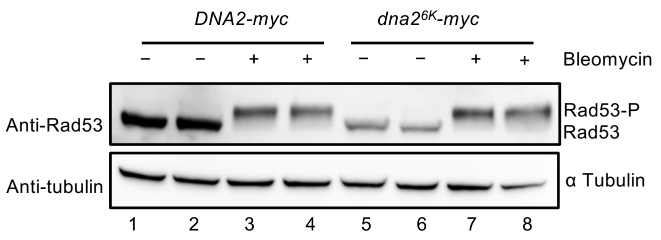

b

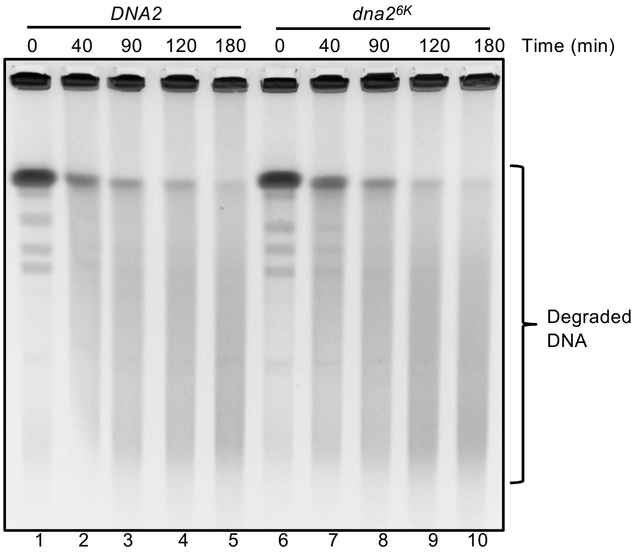

Supplementary Figure 4 Phenotypic analyses of *dna2*^{6K} and *DNA2* strains (JKM139 background). (**a**) Representative western blot analysis of extracts from synchronized wild type *DNA2* and *dna2*^{6K} cells. Rad53 phosphorylation was monitored in samples untreated or treated with 25 µg/ml bleomycin for 3 h. Two replicates are shown. (**b**) Representative agarose gel showing the analysis of genomic DNA separated by pulsed-field gel electrophoresis from wild type *DNA2* and *dna2*^{6K} cells (JKM139 background) after MMS treatment (0.03%) for 40, 90, 120 and 180 min.

Supplementary Figure 5

Uncropped images

Fig 1a

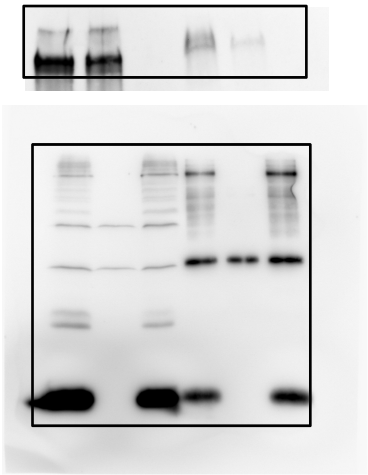

Fig 1b

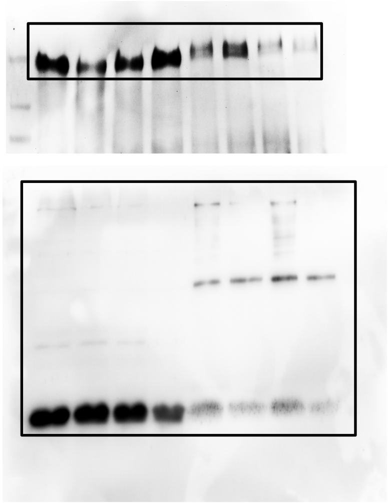

Fig 2d

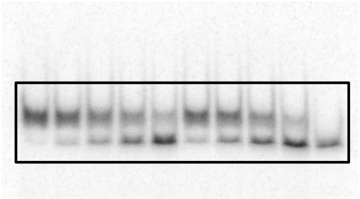

Fig 3h

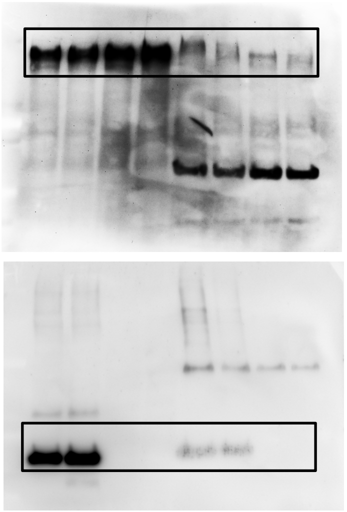

Fig 4a

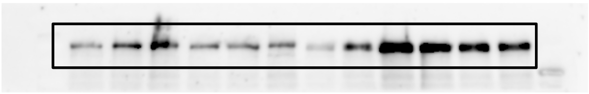

Fig 4d

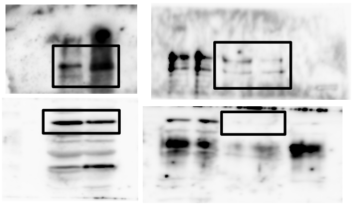

Supplementary Figure 5 Uncropped blot and gel images. The black rectangles show the final cropped part of the image from main figures (Figure 1 to 6).

Supplementary Table 1. Oligonucleotides used in this study.

Oligonucleotide	Sequence (5' – 3')	Purpose	Source
Dna2For2	CTAGAGGACGGGACACCAGA	*DNA2* forward primer for RT-PCR	This study
Dna2Rev2	CAACCAAGCCAGAAAGGCAC	*DNA2* reverse primer for RT-PCR	This study
Rad27For	GTATGCCGCAGCAAGTGAAG	*RAD27* forward primer for RT-PCR	This study
Rad27Rev	AAGGCTGTCACTGGACCAAC	*RAD27* reverse primer for RT-PCR	This study
ACT1For	GAAATGCAAACCGCTGCTCA	*ACT1* forward primer for RT-PCR	This study
ACT1Rev	TACCGGCAGATTCCAAACCC	*ACT1* forward primer for RT-PCR	This study
MAT_0kb_1	CTTGATTGTTTGCTTGAGTCTG	Preparation of DNA probe for *MAT* locus	Reference[2]
MAT_0kb_2	ACTAACAATACTTCAGTTTA	Preparation of DNA probe for *MAT* locus	Reference[2]
BUD5_3kb_1	CCAGTTATCGTCCTACGTTC	Preparation of DNA probe for *BUD5* locus	Reference[2]
BUD5_3kb_2	GGTAAGCCTTGGAACCTTAG	Preparation of DNA probe for *BUD5* locus	Reference[2]
SNT1_10kb_1	CTATCGATGGCTCTATAAGAC	Preparation of DNA probe for *SNT1* locus	Reference[2]
SNT1_10kb_2	CACGACTTATTGGACTAGTG	Preparation of DNA probe for *SNT1* locus	Reference[2]
FEN2_28kb_1	CACCAATGCATATATATCCG	Preparation of DNA probe for *FEN2* locus	Reference[2]
FEN2_28kb_2	GAATAGTCGACCAGTCTAAC	Preparation of DNA probe for *FEN2* locus	Reference[2]
Ctrl_TRA1_1	GTCCTAATACGACTTTTCAAATTG TCCTTTATGTCCGTCA	Preparation of DNA probe for *TRA1* locus	Reference[2]
Ctrl_TRA1_2	ATACTTGTAAGCACTCTTCCTGTA GTGAATATCACTTTTG	Preparation of DNA probe for *TRA1* locus	Reference[2]

PC292	GGTACTCAAGTGACGTCATAGACGATTACATTGCTAGGACATGCTGTCTAGAGACTATCGC	Preparation of DNA substrates	Reference[3]
X12-4SC	GCGATAGTCTCTAGACAGCATGTCCTAGCAA	Preparation of DNA substrates	Reference[4]
X12-3	GACGTCATAGACGATTACATTGCTAGGACATGCTGTCTAGAGACTATCGC	Preparation of DNA substrates	Reference[4]
LigFor	ACGCATTAGCTAGCGGATCCCTGGAAGTTCTGTTCCAGGGGCCCATGCGCAGATTACTGACCGGTTG	Preparation of DNA substrates	Reference[5]
LigRev	ACGCATTACTCGAGATTTTGCATGTGGGATTGGT	Preparation of DNA substrates	Reference[5]

Supplementary Table 2. Yeast strains used in this study.

Strain	Genotype	Source
FF18733	*MAT*a *his7-2 leu2-3,112 lys1-1 trp1-289 ura3-52*	F. Fabre
JKM139	*MAT*a *hoΔ hmlΔ::ADE1 hmrΔ::ADE1 ade1-100 leu2-3,112 lys5 trp1Δ::hisG ura3-52 ade3::GAL::HO*	Reference[1]
yWH436	JKM139 *pif1-m2*	Reference[2]
ML1	FF18733 *DNA2-9MYC-KanMX4*	This study
ML2	FF18733 *DNA2-9MYC-KanMX4 + YEp181-CUP1-His-SMT3*	This study
ML3	FF18733 *DNA2-9MYC-KanMX4 + YEp181*	This study
ML68	FF18733 *+ YEp181-CUP1-His-SMT3*	This study
ML69	FF18733 *+ YEp181*	This study
ML4	FF18733 *DNA2-9MYC-KanMX4 siz1::URA3 + YEp181-CUP1-His-SMT3*	This study
ML5	FF18733 *DNA2-9MYC-KanMX4 siz2::hphNT1 + YEp181-CUP1-His-SMT3*	This study
ML6	FF18733 *DNA2-9MYC-KanMX4 siz1::URA3 siz2::hphNT1 + YEp181-CUP1-His-SMT3*	This study
ML7	JKM139 *DNA2-9MYC-KanMX4*	This study
ML8	JKM139 *DNA2-9MYC-KanMX4 + YEp181-CUP1-His-SMT3*	This study
ML76	JKM139 *DNA2-9MYC-KanMX4 + YEplac181*	This study
ML9	JKM139 $dna2^{K21A\ K33A\ K60A\ K93A\ K103A\ K247A}$ ($dna2^{6K}$)	This study
ML10	JKM139 $dna2^{K21A\ K33A\ K60A\ K93A\ K103A\ K247A}$*-9MYC-KanMX4*	This study
ML11	JKM139 $dna2^{K21A\ K33A\ K60A\ K93A\ K103A\ K247A}$*-9MYC-KanMX4 + YEp181-CUP1-His-SMT3*	This study
ML12	JKM139 $dna2^{K21A\ K33A\ K60A\ K93A\ K103A\ K247A}$*-9MYC-KanMX4 + Yeplac181*	This study
ML13	yWH436 $dna2^{K21A\ K33A\ K60A\ K93A\ K103A\ K247A}$ ($dna2^{6K}$)	This study
ML14	yWH436 $dna2^{K33A\ K60A\ K93A\ K103A\ K247A}$ ($dna2^{5K}$)	This study
ML15	yWH436 $dna2^{K60A\ K93A\ K103A\ K247A}$ ($dna2^{4K}$)	This study
ML16	yWH436 $dna2^{K247A}$ ($dna2^{1K}$)	This study
ML17	yWH436 *dna2::URA3*	This study
ML18	JKM139 *exo1::hphNT1*	This study
ML20	JKM139 $dna2^{K21A\ K33A\ K60A\ K93A\ K103A\ K247A}$ ($dna2^{6K}$) *exo1::hphNT1*	This study
LR1	JKM139 *RAD27-9MYC-natNT2*	This study
W303	*MATα leu2-3,112 trp1-1 can1-100 ura3-1 ade2-1 his3-11,15*	R Rothstein
yLK354	W303 *ADE2 DNA2-YFP*	M. Lisby
yLK388	W303 *ADE2* $dna2^{6K}$*-YFP*	This study
yLK414	W303 *ADE2 DNA2-YFP RFA1-8Ala-CFP*	This study
yLK415	W303 *ADE2* $dna2^{6K}$*-YFP RFA1-8Ala-CFP*	This study

References

1. Ira G*, et al.* DNA end resection, homologous recombination and DNA damage checkpoint activation require CDK1. *Nature* **431**, 1011-1017 (2004).

2. Zhu Z, Chung WH, Shim EY, Lee SE, Ira G. Sgs1 helicase and two nucleases Dna2 and Exo1 resect DNA double-strand break ends. *Cell* **134**, 981-994 (2008).

3. Levikova M, Klaue D, Seidel R, Cejka P. Nuclease activity of Saccharomyces cerevisiae Dna2 inhibits its potent DNA helicase activity. *Proceedings of the National Academy of Sciences of the United States of America* **110**, E1992-2001 (2013).

4. Cejka P, Kowalczykowski SC. The full-length Saccharomyces cerevisiae Sgs1 protein is a vigorous DNA helicase that preferentially unwinds holliday junctions. *The Journal of biological chemistry* **285**, 8290-8301 (2010).

5. Levikova M, Pinto C, Cejka P. The motor activity of DNA2 functions as an ssDNA translocase to promote DNA end resection. *Genes Dev* **31**, 493-502 (2017).
