# Supplementary material for: Sumoylation regulates the stability and nuclease activity of Saccharomyces cerevisiae Dna2
Source: Commun Biol. 2019 May 8;2:174. doi: 10.1038/s42003-019-0428-0 (PMC6506525; doi:10.1038/s42003-019-0428-0)
Supplement: Supplementary file 2 — Description of Supplementary Data [file 42003_2019_428_MOESM2_ESM.pdf]

Description of Additional Supplementary Files

File Name: Supplementary Data 1

Description: Data file containing numerical values that were used to plots graphs from Figures 2-6.
