# Supplementary material for: Sumoylation regulates the stability and nuclease activity of Saccharomyces cerevisiae Dna2
Source: Commun Biol. 2019 May 8;2:174. doi: 10.1038/s42003-019-0428-0 (PMC6506525; doi:10.1038/s42003-019-0428-0)
Supplement: Supplementary file 4 — Reporting Summary [file 42003_2019_428_MOESM4_ESM.pdf]

nature research

Corresponding author(s): Cejka Petr

# Reporting Summary

Nature Research wishes to improve the reproducibility of the work that we publish. This form provides structure for consistency and transparency in reporting. For further information on Nature Research policies, see Authors & Referees and the Editorial Policy Checklist.

## Statistical parameters

When statistical analyses are reported, confirm that the following items are present in the relevant location (e.g. figure legend, table legend, main text, or Methods section).

| n/a | Confirmed | |
|---|---|---|
| ☐ | ☒ | The exact sample size (*n*) for each experimental group/condition, given as a discrete number and unit of measurement |
| ☒ | ☐ | An indication of whether measurements were taken from distinct samples or whether the same sample was measured repeatedly |
| ☐ | ☒ | The statistical test(s) used AND whether they are one- or two-sided<br>*Only common tests should be described solely by name; describe more complex techniques in the Methods section.* |
| ☒ | ☐ | A description of all covariates tested |
| ☒ | ☐ | A description of any assumptions or corrections, such as tests of normality and adjustment for multiple comparisons |
| ☐ | ☒ | A full description of the statistics including central tendency (e.g. means) or other basic estimates (e.g. regression coefficient) AND variation (e.g. standard deviation) or associated estimates of uncertainty (e.g. confidence intervals) |
| ☐ | ☒ | For null hypothesis testing, the test statistic (e.g. *F*, *t*, *r*) with confidence intervals, effect sizes, degrees of freedom and *P* value noted<br>*Give P values as exact values whenever suitable.* |
| ☒ | ☐ | For Bayesian analysis, information on the choice of priors and Markov chain Monte Carlo settings |
| ☒ | ☐ | For hierarchical and complex designs, identification of the appropriate level for tests and full reporting of outcomes |
| ☒ | ☐ | Estimates of effect sizes (e.g. Cohen's *d*, Pearson's *r*), indicating how they were calculated |
| ☐ | ☒ | Clearly defined error bars<br>*State explicitly what error bars represent (e.g. SD, SE, CI)* |

*Our web collection on statistics for biologists may be useful.*

## Software and code

Policy information about availability of computer code

| | |
|---|---|
| Data collection | Only commercial software was used for data collection. |
| Data analysis | Only commercial software was used for data analysis. |

For manuscripts utilizing custom algorithms or software that are central to the research but not yet described in published literature, software must be made available to editors/reviewers upon request. We strongly encourage code deposition in a community repository (e.g. GitHub). See the Nature Research guidelines for submitting code & software for further information.

## Data

Policy information about availability of data

All manuscripts must include a data availability statement. This statement should provide the following information, where applicable:

- Accession codes, unique identifiers, or web links for publicly available datasets
- A list of figures that have associated raw data
- A description of any restrictions on data availability

All relevant data are available from the corresponding author upon reasonable request.

# Field-specific reporting

Please select the best fit for your research. If you are not sure, read the appropriate sections before making your selection.

☒ Life sciences ☐ Behavioural & social sciences ☐ Ecological, evolutionary & environmental sciences

For a reference copy of the document with all sections, see [nature.com/authors/policies/ReportingSummary-flat.pdf](nature.com/authors/policies/ReportingSummary-flat.pdf)

# Life sciences study design

All studies must disclose on these points even when the disclosure is negative.

| | |
|---|---|
| Sample size | Sample size (or number of repeats) was chosen based on what is common in the field, and what was practical to do. |
| Data exclusions | In general, no data were excluded unless there was a valid reason to do so, e.g. loading control indicated unequal loading that invalidated the analysis or other technical issues (broken gels etc.). |
| Replication | The experiments were replicated several times as indicated in figure legends. Oftentimes, even within a single experiment, multiple enzyme concentrations were analyzed, or samples were compared at multiple time points. This also contributes to the robustness of the data. |
| Randomization | No randomization was done. |
| Blinding | No blinding was done. |

# Reporting for specific materials, systems and methods

## Materials & experimental systems

| n/a | Involved in the study | |
|---|---|---|
| ☐ | ☒ | Unique biological materials |
| ☐ | ☒ | Antibodies |
| ☒ | ☐ | Eukaryotic cell lines |
| ☒ | ☐ | Palaeontology |
| ☒ | ☐ | Animals and other organisms |
| ☒ | ☐ | Human research participants |

## Methods

| n/a | Involved in the study | |
|---|---|---|
| ☒ | ☐ | ChIP-seq |
| ☐ | ☒ | Flow cytometry |
| ☒ | ☐ | MRI-based neuroimaging |

## Unique biological materials

Policy information about [availability of materials](availability of materials)

| | |
|---|---|
| Obtaining unique materials | We obtained various yeast strains and further genetically modified them. The modified strains will be made available, the parental strains can be obtained from the corresponding authors of the respective studies. |

## Antibodies

| | |
|---|---|
| Antibodies used | All antibodies used are described in the text, including manufacturer, catalog number and dilutions. |
| Validation | The antibodies used were generally validated by the commercial manufacturer. Our experiments are in full agreement with the assumption that the antibodies are specific. |

# Flow Cytometry

## Plots

Confirm that:

☒ The axis labels state the marker and fluorochrome used (e.g. CD4-FITC).

☒ The axis scales are clearly visible. Include numbers along axes only for bottom left plot of group (a 'group' is an analysis of identical markers).

☐ All plots are contour plots with outliers or pseudocolor plots.

☒ A numerical value for number of cells or percentage (with statistics) is provided.

## Methodology

| | |
|---|---|
| Sample preparation | Yeast cell culture was pelleted for each analysis at respective time points. The pelleted cells were immediately fixed in 250 mM Tris pH7.5 and 70% ethanol at 4 degrees C overnight. The cells were pelleted again, supernatant was discarded and the cells were incubated with 50 mM Tris pH7.5 and 1mg/ml RNase A overnight at 37 degrees C. The cells were centrifuged and the pellet was mixed with 200 mM Tris pH7.5, 200 mM NaCl, 50 ug/ml Propidium Iodide and 80 mM magnesium chloride. The samples were briefly sonicated in 50 mM Tris pH7.5 before and analyzed using flow cytometer. |
| Instrument | CyAn ADP 9 flow cytometer (Beckman Coulter) |
| Software | Data was analyzed using FlowJo software (Treestar). |
| Cell population abundance | A minimum of 10,000 cells were analyzed for each sample. The sample contained only yeast cells. |
| Gating strategy | Cells stained with propidium iodide were gated on single cells via use of height versus area density plot. |

☐ Tick this box to confirm that a figure exemplifying the gating strategy is provided in the Supplementary Information.
