# Supplementary material for: Sumoylation regulates the stability and nuclease activity of Saccharomyces cerevisiae Dna2
Source: Commun Biol. 2019 May 8;2:174. doi: 10.1038/s42003-019-0428-0 (PMC6506525; doi:10.1038/s42003-019-0428-0)
Supplement: Supplementary file 5 — Peer Review File [file 42003_2019_428_MOESM5_ESM.pdf]

Editorial Note: This manuscript has been previously reviewed at another journal. This document only contains reviewer comments and rebuttal letters for versions considered at *Communications Biology* . Mentions of the other journal have been redacted.

REVIEWERS' COMMENTS:

Reviewer #1 (Remarks to the Author):

I had the opportunity to review this manuscript before for [redacted]. As I mentioned in my last revision, the authors were really close to answer all my points. The main issue was the apparent contradiction between elevated levels of non-sumylatable DNA2 and the in vitro function. Careful analysis by the authors showed that, in nuclear extracts, the 6K mutant is not overexpressed, but indeed the levels are slightly reduced. This key point makes the manuscript more solid. The rest of my concerns have been adequately addressed too. Thus, I support the publication of this mansucript in Communications Biology.

There were no remaining issues raised by the referees.
